# Development of a Novel Enhanced Biosensor System for Real-Time Monitoring of Fish Stress Using a Self-Assembled Monolayer

**DOI:** 10.3390/s19071518

**Published:** 2019-03-28

**Authors:** Haiyun Wu, Yuzu Fujii, Toshiki Nakano, Takafumi Arimoto, Masataka Murata, Haruto Matsumoto, Yasutoshi Yoshiura, Hitoshi Ohnuki, Hideaki Endo

**Affiliations:** 1 Graduate School of Marine Science and Technology, Tokyo University of Marine Science and Technology, Tokyo 1088477, Japan; wuhaiyun@kaiyodai.ac.jp (H.W.); bnyz0220@gmail.com (Y.F.); tarimoto@kaiyodai.ac.jp (T.A.); ohnuki@kaiyodai.ac.jp (H.O.); 2 Graduate School of Agricultural Science, Tohoku University, Sendai-shi 9808572, Japan; nakanot@bios.tohoku.ac.jp; 3 Hokkaido Industrial Technology Center, Hakodate-shi 0410801, Japan; murata@techakodate.or.jp (M.M.); matsumoto@techakodate.or.jp (H.M.); 4 National Research Institute of Fisheries and Environment of Inland Sea, Japan Fisheries Research and Education Agency, Takamatsu-shi 761-0111, Japan; yoshiura@fra.affrc.go.jp

**Keywords:** biosensor, real-time monitoring, fish, stress, self-assembled monolayer, glucose

## Abstract

Wireless biosensor systems were developed in our lab for monitoring blood glucose concentrations in fish as an indicator of fish stress. However, uniform immobilization of the enzyme on the surface of the electrode is difficult, so the sensor response is typically reduced at a range of high glucose concentrations during the stress monitoring. In this study, we attempted to enhance sensor response by using a self-assembled monolayer-immobilized enzyme. Glucose oxidase was immobilized on a working electrode modified with a self-assembled monolayer. The proposed biosensor showed a good correlation between the output current and the glucose concentration range of 10–3500 mg dL^−1^ under an optimized working condition. The dynamic measurement range of this newly developed sensor is significantly improved, especially over a high concentration range, which helps the sensor to achieve better performance in dramatic changes in the stress response of fish. In addition, we used biological samples from test fish and obtained a good correlation coefficient between the sensor output current and the glucose concentration using a conventional method. The proposed wireless biosensor system was also applied to monitor fish stress responses in real time through different stressors and to obtain some precise data that reflect real fish stress responses.

## 1. Introduction

Currently, breeding fish under a relatively overcrowded condition, and the deterioration of water quality due to residual foods and excrements, stress the fish [1,2,3,4]. As a result, the deterioration of immune function and occurrence of diseases in fish are frequent problems. Also, as a method to prevent the deterioration of the health condition from stress, the administration of chemicals can be performed. However, from a safety perspective for cultured fish, the influence of the chemicals themselves on fish should be considered [5,6,7]. Monitoring the stress response of fish and keeping track of their health status at all times is a more effective way to prevent deterioration of immune function.

Fluctuations in blood glucose are known as a fish stress indicator [8]. To date, a human clinical measurement kit using a colorimetric method has been used to measure fluctuations in blood glucose [9,10]. However, this method has many complicated operations, such as capture, anesthesia, and blood collection, and the operation itself may be a stress factor for fish. Also, since the obtained value only describes the stress response at the time of blood sampling, it is difficult to ascertain a continuous stress response.

In order to solve this problem, a wireless monitoring biosensor system for fish stress response was developed in our laboratory [11,12]. This system does not require complicated operations, such as anesthesia and blood collection. Furthermore, by using enzymes as a biometric identification element, it is possible to measure continuous glucose concentration quickly and easily. Some glucose biosensors were developed by immobilizing a glucose oxidase enzyme (GOx) on the electrode surface. When the sensor is immersed in a glucose solution, GOx specifically reacts with glucose and oxygen. Then, the glucose is decomposed into gluconolactone and hydrogen peroxide. Next, by applying a voltage of +650 mV (vs. Ag/AgCl) to the sensor, the hydrogen peroxide and the electroactive surface (Pt/Ir) result in the oxidation of hydrogen peroxide to oxygen and the reduction of the metal by the electrons. Since the number of electrons and the amount of glucose have a proportional relationship, it is based on the principle of determining the glucose concentration by measuring its output current value of the sensor. As a result, rapid and easy measurement of glucose concentration has become possible.

In general, plasma is used to measure the glucose concentration. However, the plasma contains contaminating substances, such as proteins, which may lower the sensitivity of the sensor. We have found a kind of interstitial fluid (ISF) in the vicinity of the adventitia of the eyes of fish (EISF), and it has been confirmed that it has fewer contaminants than the plasma and a good correlation with the glucose concentration in the blood, which can cover most of the variations in stress response. Because of its characteristics, the biosensor can perform better and have a longer usage time [11,13,14]. In addition, such substances as ascorbic acid, which may affect the accuracy of the senor, are almost negligible in fish [15]. Even if these substances exist in the fish, they do not change drastically due to stress, so they will not affect the measurement of the stress response. Therefore, we focused on EISF and measured the glucose concentration by inserting the sensor into this site.

However, this biosensor has a problem in that GOx on the surface of the working electrode cannot be uniformly immobilized, since physical immobilization and a crosslinking reaction were used to immobilize GOx on the working electrode, which was implemented in our laboratory. When GOx is unevenly immobilized on the surface of the working electrode, the GOx aggregate and the three-dimensional structure become unstable. As a result, the enzyme activity against the substrate (glucose) also becomes unstable, and there is a possibility that it would be difficult to perform accurate measurement of the glucose concentration, particularly in the high concentration range. However, this high range of glucose concentration is very important to stress monitoring; so, it should not be ignored. Therefore, improvement of the sensor response in the high glucose concentration range was a major task in the practical application of the sensor.

A self-assembled monolayer (SAM) is a high-density, highly oriented molecular aggregate that is formed in the process of chemisorption of organic molecules onto a substrate. It is characterized by the fact that the molecules constituting the SAM are gathered precisely by interaction and spontaneously form a regular sequence, and this process is called self-organization or self-integration [16].

A SAM can usually be formed by management of the temperature and reaction with the substrate for a certain period of time. When the substrate is immersed in the solution of the reactive molecule, the binding site of the molecule chemically reacts with the substrate and adsorbs to the substrate surface in the same direction [17,18,19,20,21]. At this time, intermolecular interactions, such as Van der Waals forces and hydrophobic interactions, occur between the adsorbed molecules, so that a monomolecular film with high density and high orientation can be formed. Although the number of adsorbed molecules increases with the passage of time, the reaction stops automatically when the gap on the substrate surface disappears.

This research aims to improve the responsiveness of the glucose biosensor by prototyping a SAM/GOx sensor that fixes the GOx via a SAM. In addition, we performed the measurement in the EISF of the tested fish (Nile tilapia: *Oreochromis niloticus*) and examined the possibility of measurement under various conditions, such as in a breeding environment.

## 2. Materials and Methods

### 2.1. Reagents

Glucose oxidase (from *Aspergillus niger*; E.C. 1.1.3.4, type VII-S; 158,900 units g^−1^), 3-Mercaptopropionic acid (MPA), N-Hydroxysuccinimide (NHS), and bovine serum albumin (BSA) were purchased from Sigma-Aldrich (St. Louis, MO, USA). EDC (1-Ethyl-3-(3-dimethylaminopropyl)-carbodiimide hydrochloride), sodium nitrate, acetic acid, 2-phenoxy ethanol, Glucose C-II Tests Wako^®^, heparin sodium, glutaraldehyde (grade I, 25% aqueous solution), and 5% Nafion^®^ dispersion solution were purchased from Wako Pure Chemical Industries (Tokyo, Japan). The 2-methacryloyloxyethyl phosphorylcholine polymer was purchased from NOF Corporation (Tokyo, Japan). All other reagents used for the experiments were of commercial or laboratory grade.

### 2.2. SAM/GOx Biosensor Preparation

**Figure 1:**
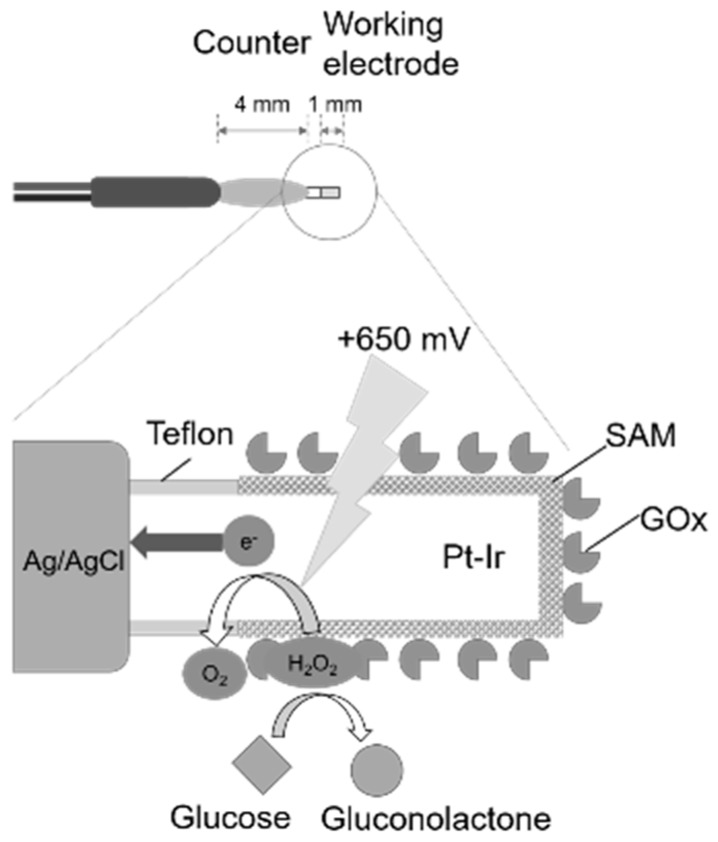
A schematic diagram of the glucose biosensors.

A small electrode for living body penetration, consisting of platinum-iridium wire (Pt-Ir) as the working electrode and silver/silver chloride (Ag/AgCl) as the counter electrode, was fabricated (Figure 1). First, 10 mm of Teflon-coated platinum-iridium wire (φ 0.178 mm, Nilaco Corporation, Tokyo, Japan) was cut out, and about 1 mm of the Teflon coating at one end was peeled off. A 70 mm lead wire (φ 0.4 mm, Unique Medical Co. Ltd., Tokyo, Japan) was joined to the electrode by soldering, and about 5 mm of the solder part was covered with heat-shrink tube (Kyowa Harmonet, Tokyo, Japan).

Subsequently, a 1 cm copper wire (φ 0.1 mm, Unique Medical Co., Ltd., Tokyo, Japan) and another 70 mm lead wire (φ 0.4 mm, Unique Medical Co., Ltd., Tokyo, Japan) were joined to the electrode by soldering. A copper wire was spirally wound around the Teflon-coated portion of the platinum-iridium wire, and a Ag/AgCl ink for a reference electrode (BAS, Tokyo, Japan) was applied to that portion and dried at 90 °C for 20 min. The Ag/AgCl ink was applied and dried twice. Finally, heat-shrink tube and Alardite^®^ (Huntsman Advanced Materials, Woodlands, TX, USA) were used for waterproofing.

Then, approximately 0.5 mm of the Teflon coat of the working electrode was peeled off, and the working electrode was immersed in a 10 mM MPA solution for 8 h to form a SAM on its surface. Next, the working electrode was immersed in 100 μL of an EDC/NHS mixed solution of 100 mg mL^−1^ for another 120 min. By this operation, the carboxyl group of the MPA terminal was substituted with a highly active ester group. Note that these operations were carried out at 25 °C.

The working electrode was immersed in the enzyme solution (250 μL, GOx: 788 units mL^−1^; BSA: 1%) for not less than 12 h to immobilize the GOx. This operation was carried out while stirring in an incubator at 4 °C. The prepared sensor was immersed in phosphate buffer (PB; 0.1 M, pH 7.8) and allowed to stand for 1 h in a refrigerator to stabilize the GOx.

The sensor was immersed in a beaker containing PB (0.1 M, pH 7.8) and subjected to ultrasonic cleaning for 10 s using an ultrasonic washer. By this operation, excess GOx that had physically adsorbed without combining with the SAM was removed.

### 2.3. Influence of Various Conditions on the Response of the SAM/GOx Sensor

#### 2.3.1. Effect of pH

The measurement temperature was set to 30 °C in the thermostatic chamber. For measurement under each condition, five stages of PB (0.1 M; pH 6.5, 7.0, 7.4, 7.8, and 8.0) were used. First, the sensor was connected to a potentiostat and immersed in 10 mL of PB (0.1 M) at each pH. Next, after the sensor was left to stand until its output current value stabilized, a standard glucose solution (5000 mg dL^−1^) was added so that the glucose concentration in PB was 200 mg dL^−1^, and the variation in the sensor output current value was recorded.

#### 2.3.2. Influence of Temperature

The measurement under each temperature condition was carried out in a thermostat chamber set at five stages: 20, 25, 30, 35, and 40 °C. First, the sensor was connected to a potentiostat and immersed in 10 mL of PB (0.1 M, pH 7.8). Next, after the sensor was left to stand until its output current value stabilized, a standard glucose solution (5000 mg dL^−1^) was added so that the glucose concentration in PB was 200 mg dL^−1^, and the variation in the sensor output current value was recorded.

### 2.4. Sensor Performance Evaluation in Buffer Solution

The measurement was carried out in a thermostat chamber set at 30 °C. The sensor was connected to a potentiostat (+650 mV) and immersed in 10 mL of PB (0.1 M, pH 7.8). After the output current value of the sensor became stable, glucose standard solution (10,000 mg dL^−1^) was added into the PB so as to achieve a glucose concentration of 10, 20, 30, 50, 70, 90, 120, 150, 200, 250, 300, 400, 500, 700, 1200, 1500, 2000, 2500, 3000, and 3500 mg dL^−1^, and the output current value was recorded.

### 2.5. Sensor Performance Evaluation in EISF

Some 2-phenoxy ethanol was dissolved in 5 L of breeding water to prepare 400 ppm of anesthetic solution. Tilapia as test fish were immersed in the solution for 5–10 min until they had become anesthetized. Next, a 27 G injection needle (Terumo, Tokyo, Japan) was attached to a 2.5 mL syringe (Terumo, Tokyo, Japan). The syringe and injection needle were filled with sodium heparin solution prepared at 3000 units mL^−1^ and then returned to the original container. Wiping off moisture near the eyeballs of the tilapia, about 1.4 mL of EISF was collected from the outer eyeball using an injection needle. Thereafter, the glucose concentration in EISF was determined using a colorimetric method (Glucose C-II Test, Wako Pure Chemical Industries, Ltd., Tokyo, Japan). The sensor was immersed in an MPC (2-methacryloyloxyethyl phosphorlycholine) polymer for 10 s then dried at room temperature for 10 min, and the operation was repeated three times. MPC is a biocompatible polymer, and it provided our biosensor with good biocompatibility with the fish. After that, 1.3 mL of collected EISF was added to a flask, and the sensor was immersed in it. After allowing the sensor’s output current value to stabilize, a standard glucose solution (5000 mg dL^−1^) was added so that the glucose concentration of the EISF became 26.87–400 mg dL^−1^, which covers the range of glucose variation in fish, and the variation in the sensor’s output current value was recorded.

### 2.6. Monitoring Stress Effects of the Different Stressors

**Figure 2:**
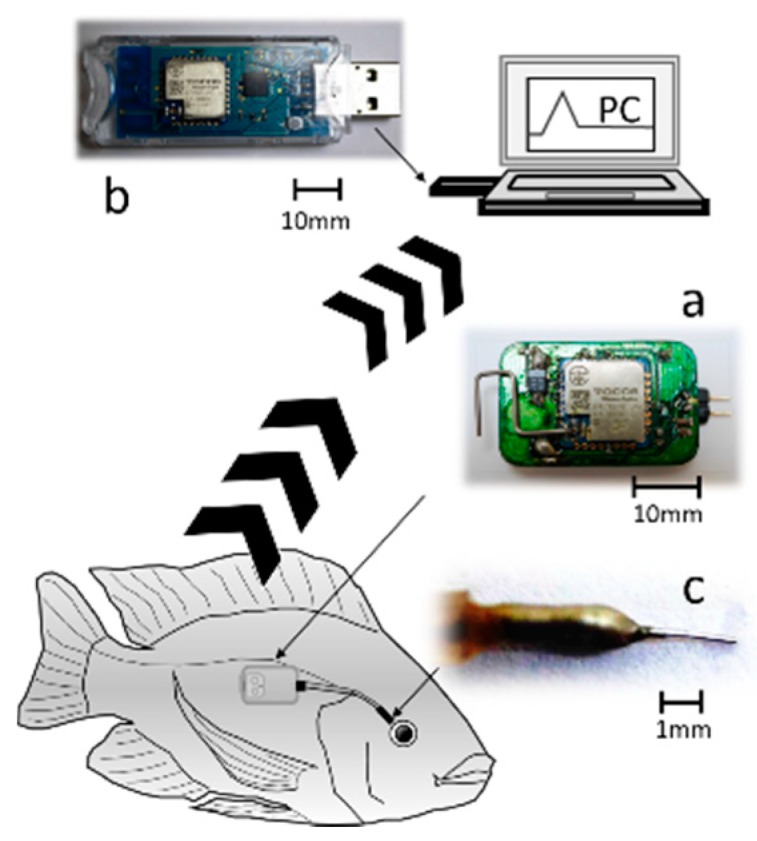
A schematic diagram of the wireless biosensor system. (**a**) The wireless transmitter: a self-made transmitter. It sends data by using radio waves. (**b**) The USB-type receiver: a self-made receiver that had been programmed to accept data from a particular transmitter. (**c**) Sensor implant site: interstitial fluid in the vicinity of the adventitia of the eyes of fish (EISF).

Nile tilapia (*Oreochromis niloticus*) were used as the test fish. The fish were cultured at the Tokyo University of Marine Science and Technology (TUMSAT) according to TUMSAT’s Guide for the Care and Use of Laboratory Animals. Each fish was anesthetized by 400 ppm 2-phenoxy ethanol to reduce its suffering when our in-house-developed radio-wave-type transmitter was attached to it. The transmitter (15 × 30 × 6 mm in size, and about 3.0 g in weight without a battery) used in this experiment was well-designed for attachment to fish. The transmitter was covered with a waterproof polypropylene sheet and further adjusted to make it more comfortably attached to the fish before the experiment. The transmitter was located on side of the fish’s body (Figure 2a), and sent signals to a receiver on land (Figure 2b). The sensor was inserted into the EISF (Figure 2c) of each fish. The depth of water in the water tank was 20 cm. After the experiment, all of the fish used in the experiment were well-cared for without any unnecessary sacrifice.

#### 2.6.1. Air Exposure

A Tilapia having a total length of 25.0 cm and a body weight of 255 g was used as a test fish. We prepared 25 L of breeding water and put it in a separate aquarium (300 × 250 × 280 mm), and kept the fish isolated for 3 days. Then, we attached the sensor to the fish the day before the measurement was taken. The measurement was started after confirming that the output current value of the sensor attached to the fish was stable. Ten minutes after the start of the measurement, the test fish was taken out of the breeding water and exposed to air for 15 min (the first blood sample was collected after the fish was exposed to air). Next, the test fish was returned to the water tank. Thereafter, blood was collected at 65 and 90 min. The glucose concentrations in the blood sample that was collected as a control were also measured using a Glucose C-II Test.

#### 2.6.2. Addition of Ammonia

A Tilapia having a total length of 22.1 cm and a body weight of 201 g was used as a test fish. We prepared 25 L of breeding water and put it in a separate aquarium (300 × 250 × 280 mm), and kept the fish isolated for 3 days. Then, we attached the sensor to the fish the day before the measurement was taken. The measurement was started after confirming that the output current value of the sensor attached to the fish was stable. Thirty minutes after the start of the measurement, the ammonia concentration in the aquarium was changed to 10 mg L^−1^, and the first blood sample was collected. Thereafter, blood was collected at 90, 160, and 230 min after the start of the experiment. The glucose concentrations in the blood sample that was collected as a control were also measured using a Glucose C-II Test.

## 3. Results and Discussion

### 3.1. Effect of Various Conditions on the Response of the SAM/GOx Sensor

The SAM/GOx sensor fabricated in this study utilizes enzymes as molecular identification elements. However, the activity of the enzyme is considered to be affected by pH and temperature. Therefore, we investigated the influence of these on the sensor response and examined the possibility of measurement under conditions similar to the actual breeding environment and EISF, assuming that the measurements were made in the EISF of the test fish.

#### 3.1.1. Effect of pH

**Figure 3:**
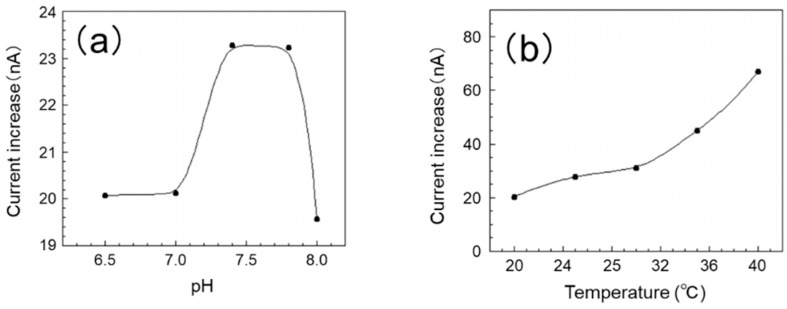
Effects of assay conditions on the sensor response. (**a**) pH (glucose concentration 200 mg dL^−1^, 30 °C); (**b**) Temperature (glucose concentration 200 mg dL^−1^, pH 7.8).

In this experiment, measurements were carried out in five stages in the pH range of 6.5 to 8.0. The effect of pH on the sensor’s output current value is shown in Figure 3a. The current increased in the pH range of 7.0 to 7.8 and then decreased gradually when the pH was over 7.8. We presumed that pH also has a great effect on the reaction, and we determined the optimum reaction pH to be 7.4–7.8. Since the EISF of tilapia (the test fish of this study) is around pH 7.8, we consider the SAM/GOx sensor to be able to measure glucose concentration even in EISF.

#### 3.1.2. Influence of Temperature

In this experiment, a measurement was carried out in five stages within the range of 20–40 °C. The influence of temperature on the sensor’s output current value is shown in Figure 3b. The response of the sensor improves as the temperature rises, and the sensor shows the highest response at 40 °C. On the other hand, it is reported that the optimum working temperature of GOx is around 50 °C. From this, we consider the optimum use temperature of this sensor to be 40 °C or greater. Here, the optimum water temperature for tilapia is 25–30 °C; however this sensor was also able to obtain a sufficient output for measurement in this range. Furthermore, within this range, a stable response was confirmed. From the above, it was found that the SAM/GOx sensor can measure the glucose concentration in the breeding environment of the test fish.

### 3.2. Response Curve and Calibration Curve of the SAM/GOx Sensor in PB

**Figure 4:**
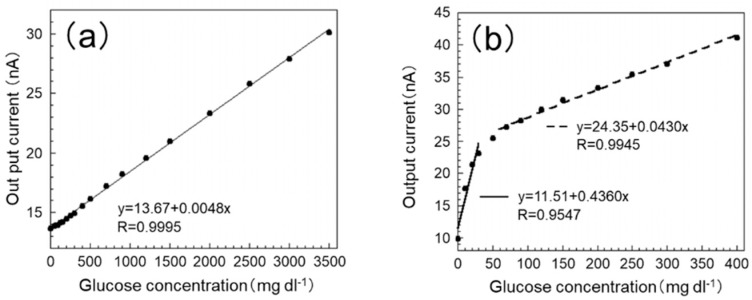
Calibration curve of the biosensor in PB. (**a**) Self-assembled monolayer/glucose oxidase enzyme (SAM/GOx) sensor; (**b**) The previous sensor assay conditions were as follows: temperature 30 °C and pH 7.8. Each sample was measured 10 min after the output current became stable. The black dots represent the relationship between the concentration of the glucose and the output current.

The calibration curves of the sensor are shown in Figure 4. From Figure 4a, it can be seen that the output current value of the proposed SAM/GOx sensor has good correlation (y = 13.67 + 0.0048x, R = 0.9995) within the range of 0–3500 mg dL^−1^ in PB. Also, from Figure 4b, it can be seen that the output current value of the GOx sensor used our previous production method [11] in PB also has good correlation (0–50 mg dL^−1^: y = 11.51 + 0.4360 x, R = 0.9547; 70 to 400 mg dL^−1^: y = 24.35 + 0.0430 x, R = 0.9945). The blood glucose level of the test fish is around 50 mg dL^−1^ and the glucose level at the time of stress loading is around 150 mg dL^−1^ generally [22]. From these results, it can be considered that both the GOx sensor and the SAM/GOx sensor can measure within the fluctuation range of the blood glucose level of the test fish.

However, it can be seen from the figure that the slope of the GOx sensor decreases from around 50 mg dL^−1^ in concentration in PB. It is considered that this is because the amount of glucose (substrate) relative to GOx approaches the saturation point after the glucose concentration of 50 mg dL^−1^. On the other hand, the SAM/GOx sensor was able to obtain a stable sensor response within the range of 0–3500 mg dL^−1^. This is considered to be due to the fact that GOx can be uniformly immobilized by using a SAM, and an efficient and stable enzymatic reaction has been realized.

As mentioned above, the blood glucose level of the test fish under stress is around 150 mg dL^−1^. If a high sensor response can be obtained in a concentration range higher than this concentration, the glucose concentration can be measured more accurately, so that the degree of stress can be more clearly grasped. Moreover, one continuous calibration curve can be obtained in the concentration range of 30–200 mg dL^−1^, which sufficiently covers the fluctuation range of the blood glucose level, instead of using different expressions at a low concentration and a high concentration.

From the above results, the SAM/GOx sensor showed high responsiveness even at high concentrations, and since it was possible to obtain a single calibration curve within the glucose concentration range of 0–3500 mg dL^−1^, it was confirmed by measurement of the stress of the test fish. It can be said that it is a more suitable sensor for fish stress monitoring.

### 3.3. Response Curve and Calibration Curve of SAM/GOx Sensor in EISF

**Figure 5:**
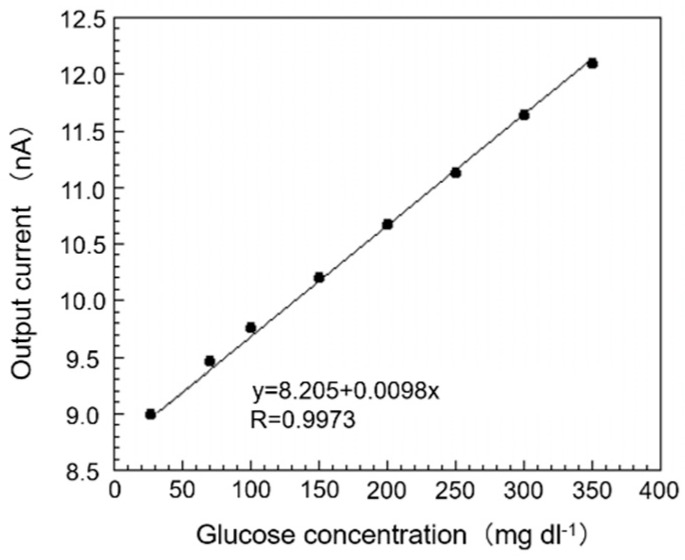
The calibration curve of the proposed biosensor in EISF. The assay conditions were as follows: temperature 30 °C. Each sample was measured 10 min after the output current became stable.

The glucose concentration in EISF of the sampled fish collected was 26.87 mg dL^−1^. Then, we added glucose standard solution (5000 mg dL^−1^) into the EISF sample so that the glucose concentration in EISF, in which the SAM/GOx sensor was immersed, became 26.87–400 mg dL^−1^. A calibration curve prepared based on this response curve is shown in Figure 5. From this figure, it can be seen that it is possible to obtain a single calibration curve within the range of the concentration of 26.87–400 mg dL^−1^ in the EISF for the output current value of the SAM/GOx sensor, and good correlation (y = 8.205 + 0.0098 x, R = 0.9973) was confirmed. From this result, it is possible to obtain a good correlation between the output current value of the sensor and the glucose concentration with little difference between the SAM/GOx sensor and the PB even in EISF, and it is possible to quantify the amount of glucose in the biological sample. Also, the sensor still holds an output value of 93.7% even after 48 h of immersion in the EISF, which is sufficient for a normal monitoring request.

### 3.4. Fish Stress Response Monitoring

#### 3.4.1. Air Exposure

**Figure 6:**
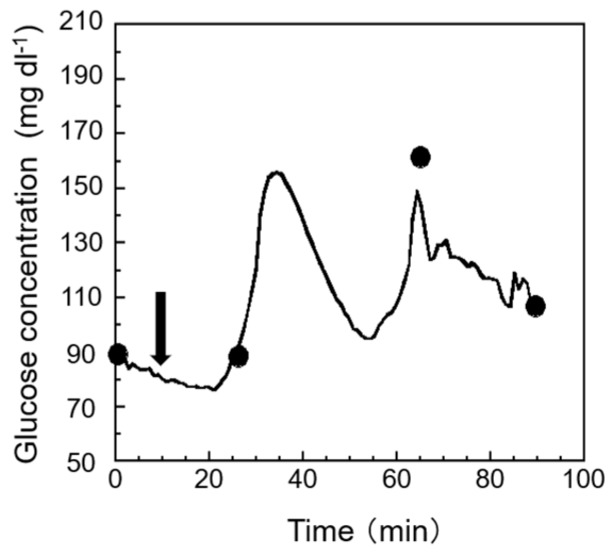
Monitoring stress response to air exposure. Glucose concentrations in the EISF, plotted as the first black circles, were used for the one-point calibration method. All other black circles indicate the blood glucose concentration that is described in Section 2.6.1. The arrows in the figure indicate points where air exposure (15 min) has started.

For terrestrial organisms, the relatively high concentration of oxygen means that oxygen is likely to be limited only during periods of intense demand; in general, activity is more likely to be limited by food than by the oxygen supply. Oxygen, however, is much more likely to be limited for aquatic organisms than for terrestrial organisms. When the oxygen supply is insufficient to meet the minimal energy demands of essential functions, suffocation occurs, and stress arises [23]. So, it is important that, when monitoring fish stress, we monitor hypoxia due to a lack of oxygen in handing or transportation in real-time. The results of the measurement of stress response by air exposure are shown in Figure 6. From the figure, it is possible to confirm the rapid rise in glucose of the test fish immediately after air exposure. In addition, a good correlation was observed between the measured value by the sensor and the conventional method. As a result, we can see that the proposed biosensor system shows a great response to reflect the change in the glucose in the EISF. Compared with other research, a similar trend was found in [24]. Compared with the conventional method (point-to-point measurement), our proposed biosensor system can provide real-time monitoring of fish stress, which can help us to quickly obtain an understanding on how to handle stress in a precise and easy way.

#### 3.4.2. Addition of Ammonia

**Figure 7:**
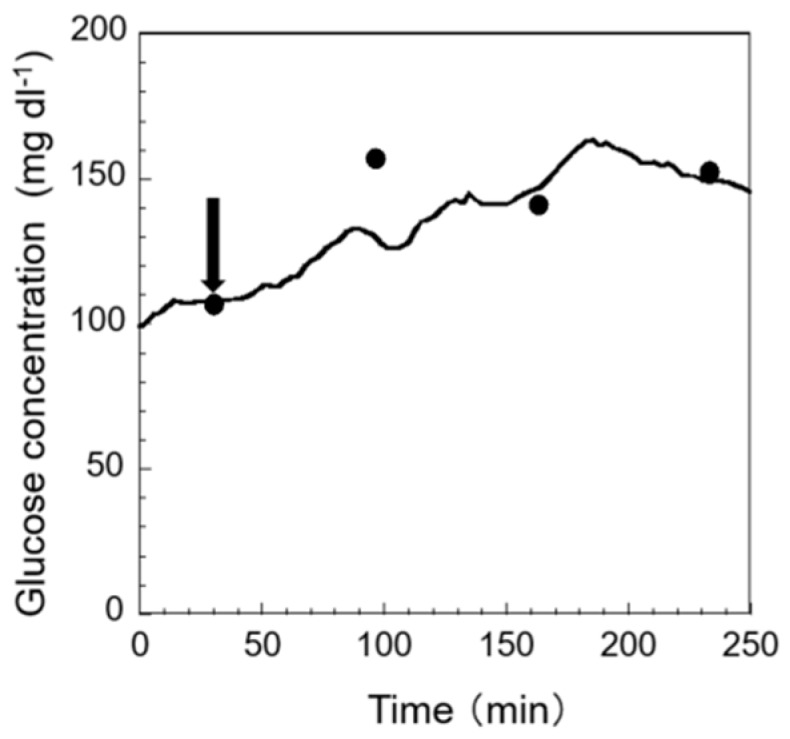
Monitoring stress response to ammonia addition. Glucose concentrations in the blood, plotted as the first black circles, were used for the one-point calibration method. All other black circles indicate the blood glucose concentration that is described in Section 2.6.2. The arrow marks the time at which the ammonia concentration changed from 0.25 mg L^−1^ to 10 mg L^−1^.

Short-term exposure to an elevated ammonia concentration in fish leads to increased gill ventilation, erratic and quick movements, a lack of foraging, and even mortality [25,26]. This kind of stress is difficult to observe unless precise long-term monitoring is performed. Through this kind of monitoring, we can obtain a deeper understanding on the status of fish more intuitively and more quickly. The results of monitoring stress response to ammonia exposure are shown in Figure 7. The arrows in the figure indicate the points where ammonia exposure has started. As shown in the figure, after exposing the fish’s body to ammonia-containing breeding water, a gradual increase in glucose concentration in EISF was observed. A similar result was also obtained in previous research [27]. From these facts, the possibility of monitoring stress response to ammonia exposure in fish using this sensor system can be suggested. As a result, the proposed sensor system can be used to monitor a chronic change in some chemical stressors.

Compared with our previous sensor’s performance, we can see that this sensor has better sensing accuracy [11]. That is to say, the calibrated glucose value from the output of the sensor is closer to the actual glucose concentration value. We think that this precision is due to the new enzyme immobilization method, which makes the surface of the sensor more able to detect the glucose concentration. This will help us to obtain a better understanding of stress response in fish.

## 4. Conclusions

In this study, we fabricated a SAM/GOx sensor using a self-assembled monolayer for the immobilization of GOx for the purpose of improving the responsiveness of a biosensor for measuring the blood glucose concentration in fishes. That is, by utilizing the characteristics of SAMs that spontaneously assemble to form a regular array, GOx was uniformly immobilized, and attempts were made to stabilize and enhance the enzymatic reaction.

The influence of various conditions (pH, temperature) on the response of this sensor was investigated. As a result, it was found that the most efficient enzyme reaction was performed at a pH of 7.4–7.8 and a temperature of 40 °C or higher. On the other hand, it was found that the glucose concentration can also be measured in the EISF of the test fish (around pH 7.8) and in the breeding environment of the test fish (25 °C to 30 °C). In order to confirm the response of this sensor, a measurement was carried out in a phosphate buffer solution (0.1 M, pH 7.8), and the relationship between the sensor’s output current value and the glucose concentration was examined. As a result, a good correlation was observed between the output current value and the glucose concentration within the range of the glucose concentration of 0–3500 mg dL^−1^. This concentration range was much wider than that of the former GOx sensor. From these results, it was confirmed that the SAM/GOx sensor is a highly responsive glucose biosensor for fish stress measurement in a wide concentration range. With the application of this sensor to a biological sample, a measurement was carried out in EISF sampled from the test fish, and the relationship between the sensor’s output current value and the glucose concentration was examined. As a result, we found a good correlation between the output current value and the glucose concentration within the range of glucose concentration of 0–400 mg dL^−1^, which sufficiently covered the fluctuation range of the blood glucose level of the test fish, and one continuous calibration curve could also be obtained. As a result, the application of the SAM/GOx sensor to biological samples was confirmed. In this paper, we also evaluated whether, if the environment was changed to an unbearable point, fish will feel stress and our sensor system would reflect this stress from the variation in glucose concentration. We found that our proposed sensor system has a very good response to changes in stress levels even in real free-swimming fish.
